# Knockout of Lysosomal Enzyme-Targeting Gene Causes Abnormalities in Mouse Pup Isolation Calls

**DOI:** 10.3389/fnbeh.2016.00237

**Published:** 2017-01-04

**Authors:** Terra D. Barnes, Timothy E. Holy

**Affiliations:** Department of Neuroscience, Washington University in St. Louis School of MedicineSt. Louis, MO, USA

**Keywords:** mouse ultrasonic vocalizations, lysosomal enzymes, Gnptab, mucolipidosis II, lysosomal enzyme trafficking pathway

## Abstract

Humans lacking a working copy of the *GNPTAB* gene suffer from the metabolic disease Mucolipidosis type II (MLII). MLII symptoms include mental retardation, skeletal deformities and cartilage defects as well as a speech delay with most subjects unable to utter single words (Otomo et al., 2009; Cathey et al., 2010; Leroy et al., 2012). Here we asked whether mice lacking a copy of Gnptab gene exhibited vocal abnormities. We recorded ultrasonic vocalizations from 5 to 8 day old mice separated from their mother and littermates. Although *Gnptab*^−/−^ pups emitted a similar number of calls, several features of the calls were different from their wild type littermates. *Gnptab*^−/−^ mice showed a decrease in the length of calls, an increase in the intra-bout pause duration, significantly fewer pitch jumps with smaller mean size, and an increase in the number of isolated calls. In addition, *Gnptab*^−/−^ mice vocalizations had less power, particularly in the higher frequencies. *Gnptab*^+/−^ mouse vocalizations did not appear to be affected. We then attempted to classify these recordings using these features to determine the genotype of the animal. We were able to correctly identify 87% of the recordings as either *Gnptab*^−/−^ or *Gnptab*^+/+^ pup, significantly better than chance, demonstrating that genotype is a strong predictor of vocalization phenotype. These data show that deletion of genes in the lysosomal enzyme targeting pathway affect mouse pup isolation calls.

## Introduction

Mucolipidosis II/III are diseases caused by a knockout or abnormities in the N-acetylglucosamine-1-phosphate transferase alpha and beta subunits (GNPTAB) gene. GNPTAB codes for the catalytic α/β subunit of lysosomal enzyme targeting pathway (LETP) enzyme *N*-acetylglucosamine-1-phosphotransferase (GlcNAc-1-phosphotransferase) (Kornfeld, 1987). This enzyme is the main enzyme in the lysosomal enzyme trafficking pathway (LETP). GlcNAc-1-phosphotransferase adds a mannose 6-phosphate targeting signal which is recognized by mannose 6-phosphate receptor. Mucolipidosis II is caused by near total absence of phosphotransferase activity resulting from homozygosity or compound heterozygosity for frameshift or nonsense mutations (Cathey et al., 2010). Patients suffer from craniofacial and orthopedic manifestations as well as speech, ambulation, and cognitive impairments (Cathey et al., 2010). Mucolipidosis III is a milder form of the disease caused by less severe mutations in GNPTAB or the gene that codes for the gamma subunit of N-acetylglucosamine-1-phosphate transferase, GNPTG.

Mucolipidosis is not the only lysosomal enzyme sorting disorder associated with speech abnormalities. Often the first symptom of late onset Tay Sachs disease is stuttering (Philippart et al., 1995; MacQueen et al., 1998; Shapiro and Natowicz, 2009). Symptoms of gangliosidsis, a disease caused by mutations in the gene encoding lysosomal beta-galactosidase, include a severe, sometimes progressive stutter (Lichtenberg et al., 1988; Nardocci et al., 1993; Chakraborty et al., 1994). Other lysosomal enzyme diseases, including Salla disease and Pompe disease, appear to cause other speech abnormalities (Aula et al., 1979; Morse et al., 2005; van Gelder et al., 2012).

A mouse model of Mucolipidosis with a knockout of GNPTAB has been identified and studied (Gelfman et al., 2007; Idol et al., 2014; Paton et al., 2014). *Gnptab*^−/−^ mice have been found to have progressive neurodegeneration including neuronal loss, astrocytosis, microgliosis and Purkinje cell depletion that was evident as early as 4 months (Idol et al., 2014). *Gnptab*^−/−^ mice were found to have a total loss of acid hydrolase phosphorylation, which results in depletion of acid hydrolases in mesenchymal-derived cells (Gelfman et al., 2007; Idol et al., 2014; Paton et al., 2014). Behaviorally, *Gnptab*^−/−^ mice, at 1 month of age, were found to perform normally on a 1 h locomotor activity test, but were found to be behaviorally impaired on tests such as how long mice remained on an elevated platform or accelerating rotorod, how long mice took to climb down a pole, and other sensory motor tests (Idol et al., 2014; Paton et al., 2014).

Surprisingly, stuttering can be caused by different mutations in the same *GNPTAB* gene as well as the other two genes that make up the enzymes in the lysosomal enzyme targeting pathway. The behavior of mice engineered to carry this stuttering associated mutation in *Gnptab* has been extensively studied, particularly their vocalizations (Barnes et al., 2016).

Mice produce ultrasonic vocalizations (USV) in a range of social situations. These vocalizations have been characterized as “songs” as they have repeated calls and complex structure (Holy and Guo, 2005). One type of innate mouse vocalization is the isolation call of pups. Mouse pups, when separated from their mother during the first 2 weeks of life, spontaneously vocalize (Ehret, 1992). The pup isolation calls of the *Gnptab*^mut/mut^ mice were recorded and analyzed. The number of vocalizations per unit of time, the length of pauses and the temporal entropy of the USV were compared to wildtype littermates and found to be abnormal. A battery of other behavioral tests were done and found to be normal. The abnormalities found in the mouse pup isolated calls of these mice were then compared to that of people who stutter that have a mutation in the same pathway. The abnormalities were reminiscent of human stuttering (Barnes et al., 2016).

Here, we ask whether *Gnptab*^−/−^ differed from *Gnptab*^+/+^ in their USVs. This innate complex motor behavior manifests at an early age. We were therefore able to study the behavioral abilities of the *Gnptab*^−/−^ mice earlier than previous studies on this model of mucolipidosis – on postnatal day 5 (P5) and 8 (P8). We find that *Gnptab*^−/−^ mice pups calls were grossly normal; they vocalized, and produced call waveforms that were similar to those of wildtype mice. On closer inspection, we found several abnormal features of *Gnptab*^−/−^ calls: shorter calls, longer inter-call intervals, more isolated calls, and fewer abrupt pitch jumps. These data show that this mutation—a deletion—in the lysosomal enzyme targeting pathway affects mouse pup isolation calls, reminiscent of the effect of point mutations in mice and humans. These data support the conclusion that there is a commonly disrupted pathway in both species. Furthermore, this is the earliest demonstration of a behavioral deficit in this model of Mucolipidosis II/III.

## Results

Mice were bred on a 129/SvEvBrd and C57BL/6J background. On P5 and P8, pups were isolated from their dam in and vocalizations recorded for three and half minutes. Mice were then genotyped and analyzed using custom MATLAB software. Syllable boundaries and identification were done by an automated algorithm.

Heterozygous parents of patients with Mucolipidosis II/III (*Gnptab*^+/−^) have no apparent phenotype. We therefore hypothesized that there would be no difference in the *Gnptab*^+/−^*, mice* heterozygous for the knockout. Supplemental Table 1 shows that this was indeed the case. Across all of our measurements, we found no significant difference between the heterozygous mice vocalizations and the wildtype mice vocalizations. We therefore first concentrate our report on the data from the wild type mice and homozygous knockout.

### Rate and duration of vocalizing

**Figure 1:**
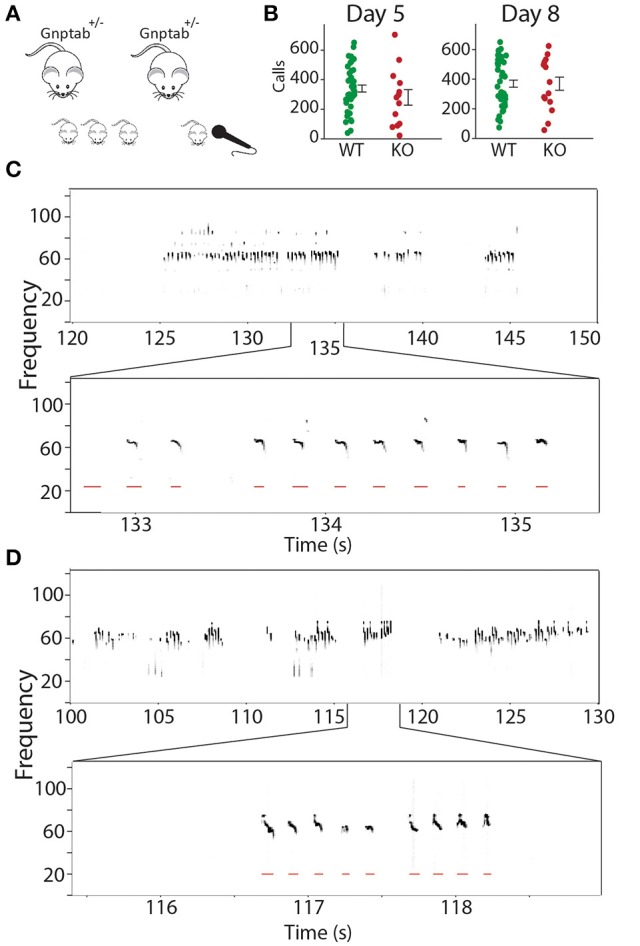
**Recording mouse pup vocalizations. (A)** Mice heterozygous for the knockout of *Gnptab* were bred. On P5 and P8, pup isolation calls from their offspring (*Gnptab*^+/+^, *Gnptab*^+/−^ *Gnptab*^−/−^ were recorded. **(B)** The number of calls for *Gnptab*^+/+^ (WT, green, P5, *n* = 40; P8, *n* = 41) and *Gnptab*^−/−^ pups (KO, red, P5, *n* = 13; P8, *n* = 15) on P5 (left) and P8 (right). The differences between the two genotypes were not significant on either day. Each dot represents the mean for one animal. Bars indicate the standard error of the mean. **(C)** Top, spectrogram of pup isolation calls recorded from a wild-type P8 *Gnptab*^+/+^ pup. Below is a zoomed in version showing detected calls in red. **(D)** Top, spectrogram of pup isolation calls recorded from a knockout P8 *Gnptab*^−/−^ pup. Below is a zoomed in version showing detected calls in red.

**Figure 2:**
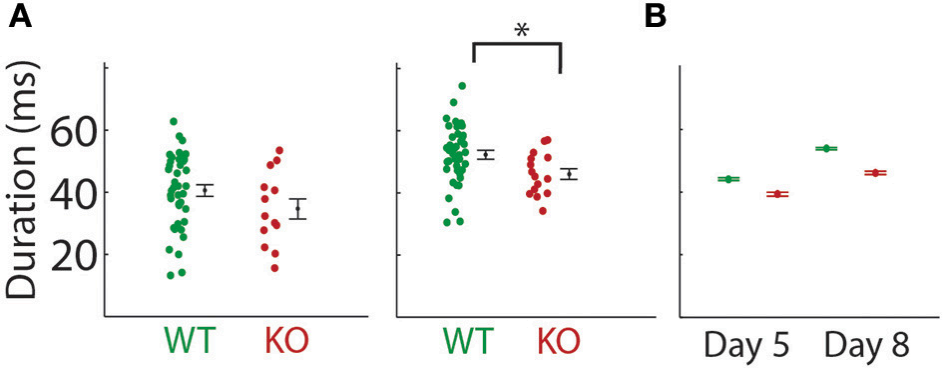
**Duration of the mouse pup isolation calls were decreased in the ***Gnptab***^**−/−**^ mice. (A)** Duration of calls emitted by pups on P5 (left) and P8 (middle) by *Gnptab*^+/+^ (green) and *Gnptab*^−/−^ pups. Each dot represents the mean for one animal. Note there was a significant difference between *Gnptab*^+/+^ mean call duration and *Gnptab*^−/−^ mean call duration on P8 (*t*-test; *p* = 0.024). Error bars indicate standard error of the mean **(B)** Duration of calls analyzed treating each vocalizations, rather than animal, as a separate data point, on P5, WT (green, 13624 calls, *n* = 40), and KO (red, 3641 calls, *n* = 13) and P8 (WT (green, 15172 calls, *p* = 41), and KO (red, 5548 calls, *n* = 15). Error bars indicate 95% confidence intervals. Analyzed this way, groups were significantly different on P5 and P8 (*t*-test; *p* ≤ 0.000002). Asterisks denote *p* < 0.05.

We recorded pup isolation calls from *Gnptab*^−/−^ on P5 and P8 and compared them to wild type littermates *Gnptab*^+/+^ (Figures 1A,B). *Gnptab*^−/−^ vocalized were found to emit pup isolation calls. Their calls included bouts of vocalization as well as high, low and pitch jump calls. No differences were found in either the number of calls (P5; *t*-test; *t* = 1.154, *p* = 0.254, WT, *n* = 40; KO, *n* = 13, P8; *t*-test; *t* = 0.0004, *p* = 0.997, WT, *n* = 41; KO *n* = 15 Figure 1C), or the number of clicks (short broadband noise pulses) (P5; *t*-test; *t* = 0.820, *p* = 0.416, P8; *t*-test; *t* = 1.065, *p* = 0.291) emitted by mice with the two different genotypes. Further analysis revealed a significant difference in the mean duration of the calls emitted by the *Gnptab*^−/−^ pups (0.046 ± 0.002 s) compared to the *Gnptab*^+/+^ pups (0.052 ± 0.002 s) at P8 (*t*-test; t = 2.332, *p* = 0.024; ~88% of wild type duration, Figure 2A). This is analysis was performed by taking the mean duration of all calls for each animal and comparing them across groups. A more sensitive measurement, wherein each call duration is averaged over the genotype instead of over the animal (which increases *n* considerably and can therefore pick up smaller differences) shows that this difference is present in P5 recordings as well (Figure 2B).

### Pauses in vocalizing

Next the pauses in the pup vocalizations were analyzed. Not unlike human vocalizations where there are time periods of quietness between sentences or thoughts and shorter brief pauses between words or phrases, pup song contains long pauses, called inter-bout pauses interspersed with bouts of calls. This can be seen in the example sonogram in Figure 1B and in the long tail of the histogram showing pause durations (Supplemental Figures 1A–C). There are also brief pauses, termed intra-bout pauses, between calls during bouts.

**Figure 3:**
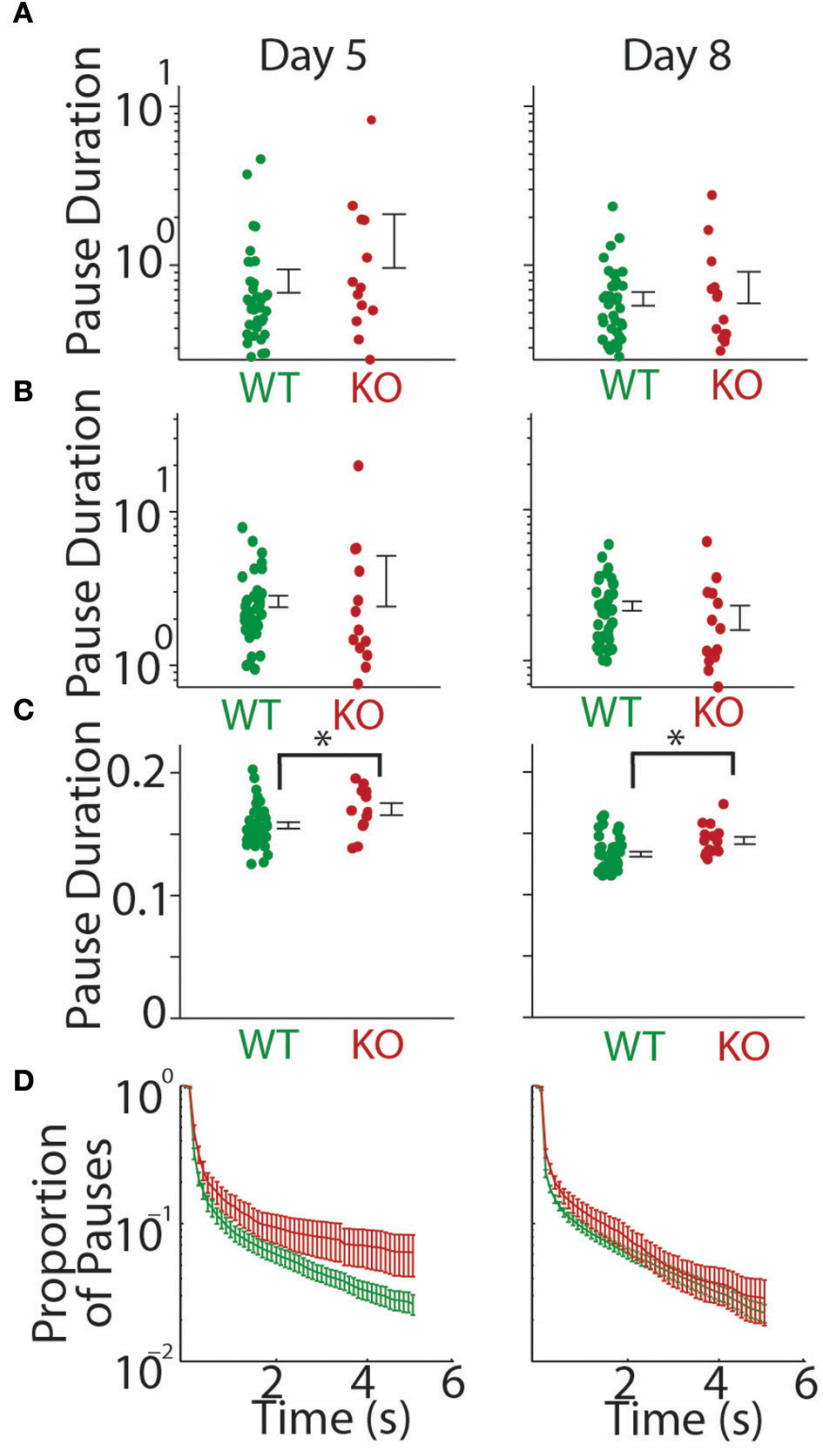
**Duration of the mouse pup pauses increased in ***Gnptab***^**−/−**^ mice. (A)** Duration of all pauses by *Gnptab*^+/+^ (green) pups and *Gnptab*^−/−^ pups (red) on P5 (left) and P8 (right). Each dot represents the mean of one animal. Error bars represent standard error of the mean. Note there was no significant difference between the two genotypes though there was a trend in P5 (*t*-test; *p* = 0.084). **(B)** Same as in **(A)** but for inter-bout pause duration. **(C)** Same as in A but intra-bout pause duration. Note the significant difference between *Gnptab*^+/+^, and *Gnptab*^−/−^ animals on P5 (*t*-test, *p* = 0.018) and P8 (*t*-test; *p* = 0.006). Asterisks denote *p* < 0.05. **(D)** Proportion of long pauses with a steadily increasing criterion for “long pauses.” Data on a log scale.

We first looked at all pauses (both inter-bout and intra-bout pauses). We found no significant difference in the mean pause duration at P5 (WT = 0.806 ± 0.135 s, KO = 1.526 ± 0.566 s, *t*-test; *t* = 1.765, *p* = 0.084) and P8 (WT = 0.615 ± 0.061 s, KO = 0.739 ± 0.166 s, *t*-test; *t* = 0.854, *p* = 0.397, Figure 3A). We next separately analyzed inter-bout pause durations and intra-bout pause durations. Inter-bout pause durations were not significantly different on P5 (WT = 2.61 ± 0.228 s, KO = 3.78 ± 1.37 s, *t*-test; *t* = 1.308, *p* = 0.197) or P8 (WT = 2.31 ± 0.169 s, KO = 1.96 ± 0.360 s, *t*-test; *t* = 0.974, *p* = 0.334, Figure 3B). Analyzing the intra-bout pause durations (inter- call interval in a series of calls), *Gnptab*^−/−^ mice showed a significantly increased duration of intra-bout pause compared to *Gnptab*^+/+^ on P5 (WT = 0.157 ± 0.003 s, KO = 0.170 ± 0.005 s, *t*-test; *t* = 2.442, *p* = 0.018) and P8 (WT = 0.133 ± 0.002 s, KO = 0.144 ± 0.003 s, *t*-test; *t* = 2.841, *p* = 0.006, Figure 3C). The proportion of large pauses in the *Gnptab*^−/−^ was increased for a subset of pause lengths compared to *Gnptab*^+/+^. This can be seen in Figure 3D where the criterion for large pauses steadily increases along the *x*-axis.

### Sound pressure

**Figure 4:**
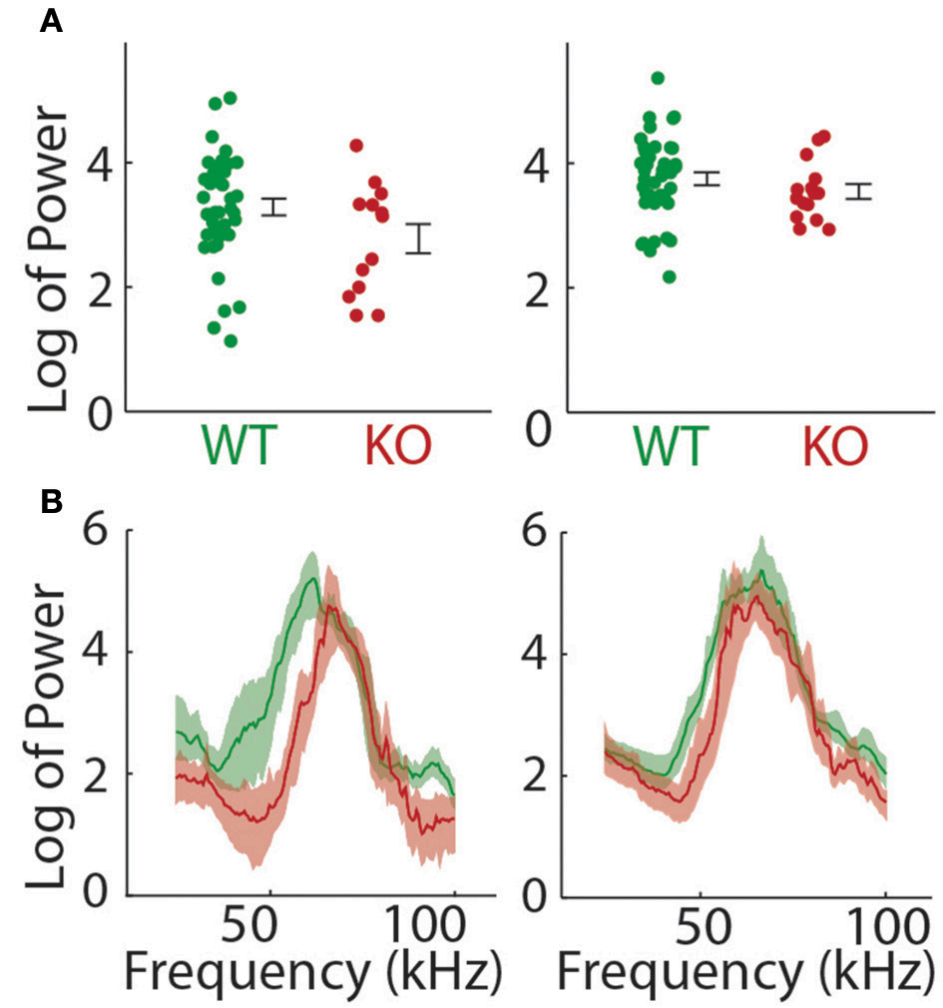
**Power in pup isolation calls. (A)** Mean power across all frequencies in *Gnptab*^+/+^ pups (Green) compared to *Gnptab*^−/−^ (red) in P5 (*t*-test, *p* = 0.076, left) and P8 *t*-test, *p* = 0.292 (right). Note that overall, there was no significant difference in the level of power in the calls of mice with different genotypes. **(B)** Power spectrum of calls in the *Gnptab*^−/−^ pups compared to the *Gnptab*^+/−^ and *Gnptab*^+/+^ pups. Shaded areas represent 95% confidence intervals.

We next examined the power spectrum of the vocalizations to see if there was any systematic difference in the pitch or loudness of the calls made by each genotype that could explain some of the findings above. The total power summed over all frequency bands was not significantly different between groups on either day of recording (*t*-test; P5, *t* = 1.809, *p* = 0.0764, P8, *t* = 1.063, *p* = 0.2923; Figure 4A). The power spectrum of the pup isolation calls is shown in Figure 4B. When broken down by frequencies, we found a significant decrease in the *Gnptab*^−/−^ compared to wildtype for the power in all frequency bands except 65–85 KHz range on P5. On P8, we found significant decreases in *Gnptab*^−/−^ compared to wildtype in the 35–65 KHz range and in the 80–100 KHz range.

### *Gnptab*^−/−^ pups: weight and vocalization

**Figure 5:**
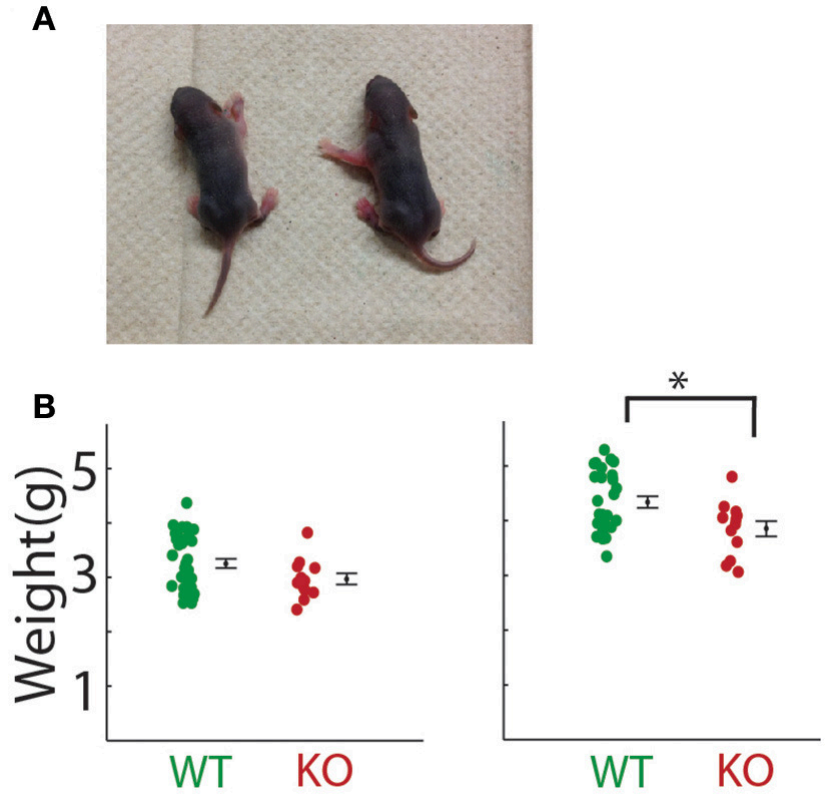
**Physical attributes of pups. (A)** Photo of pups on P8. *Gnptab*^−/−^ is on the left; the right pup is *Gnptab*^+/+^. **(B)** Weight of pups on day P5 (left) and day P8 (right) of *Gnptab*^+/+^ (WT; green) and *Gnptab*^−/−^ (KO; red). On P5, there was a non-significant trend toward *Gnptab*^−/−^ pups weighing less than *Gnptab*^+/+^ pups (*t*-test; *p* = 0.090). On P8, *Gnptab*^−/−^ pups weighed less than *Gnptab*^+/+^ pups (*t*-test; *p* = 0.015). Bars indicate the standard error of the mean. Asterisks denote *p* < 0.05.

We also tested whether the alterations in *Gnptab*^−/−^ vocalizations might be might be attributed to changes in overall physical health. *Gnptab*^−/−^ pups gross appearance was normal (Figure 5A). However, P5 *Gnptab*^−/−^ animals showed a tendency toward slightly reduced body weight (WT = 3.25 ± 0.09 grams, KO = 2.97 ± 0.10 grams; *t*-test; *t* = 1.734, *p* = 0.09). By P8, this difference was significant (WT = 4.34 ± 0.11 grams, KO, 3.85 ± 0.14 grams; *t*-test; *t* = 2.554, *p* = 0.015, Figure 5B). This is consistent with previous reports (Gelfman et al., 2007; Idol et al., 2014). Due to this significant difference in weight between the *Gnptab*^+/+^ and *Gnptab*^−/−^ pups on P8, we performed a regression analysis of *Gnptab*^+/+^ pups for each of our parameters to determine if they directly correlate with the weight of the animal (Table 1).

**Table 1:** **Correlation of weight and features of pup calls for ***Gnptab***^**+/+**^ and ***Gnptab***^**−/−**^ mice on P5 and P8**.

	***Gnptab***^**+/+**^				**All**				***Gnptab***^**−/−**^			
	**P5**		**P8**		**P5**		**P8**		**P5**		**P8**	
	**R**	***P*****-Value**	**R**	***P*****-Value**	**R**	***P*****-Value**	**R**	***P*****-Value**	**R**	***P*****-Value**	**R**	***P*****-Value**
Call duration	−0.001	0.994	−0.178	0.375	0.254	0.031	0.001	0.996	0.688	0.013^*^	0.433	0.160
Intra bout pause duration	−0.283	0.089^x^	−0.245	0.218	−0.454	0.00006^*^	−0.350	0.00490^*^	−0.837	0.0007^*^	−0.539	0.071^x^
Number of bouts	0.463	0.004^*^	0.135	0.503	0.355	0.002^*^	−0.096	0.452	0.211	0.510	0.103	0.7508
Percent isolated calls	0.120	0.478	0.453	0.0176^*^	−0.188	0.113	0.196	0.124	−0.629	0.028^*^	0.321	0.310
Mean calls per bout	−0.294	0.078^x^	−0.488	0.009^*^	−0.002	0.989	−0.243	0.0548^x^	0.390	0.209	−0.361	0.248
Power	0.128	0.451	0.058	0.773	−0.105	0.381	0.043	0.737	0.287	0.366	−0.630	0.028^*^
Pitch jumps (5 KHz)	−0.035	0.836	−0.079	0.694	0.057	0.634	0.010	0.938	−0.102	0.752	−0.211	0.511
Max size of pitch jumps	−0.058	0.731	−0.083	0.68	0.048	0.690	−0.008	0.680	−0.158	0.624	−0.286	0.368

*Asterisks denote p < 0.05, x denotes p < 0.1*.

We found that for intra-bout pause duration (P5), number of bouts (P5), percent of isolated calls (P8), and mean calls per bout (P8), there was a significant correlation. Note that there was also a trend in the mean calls per bout on P5 making this variable the most likely to be explained by weight differences. The explanatory power if each variable can be seen in Table 1.

### Pitch jumps

**Figure 6:**
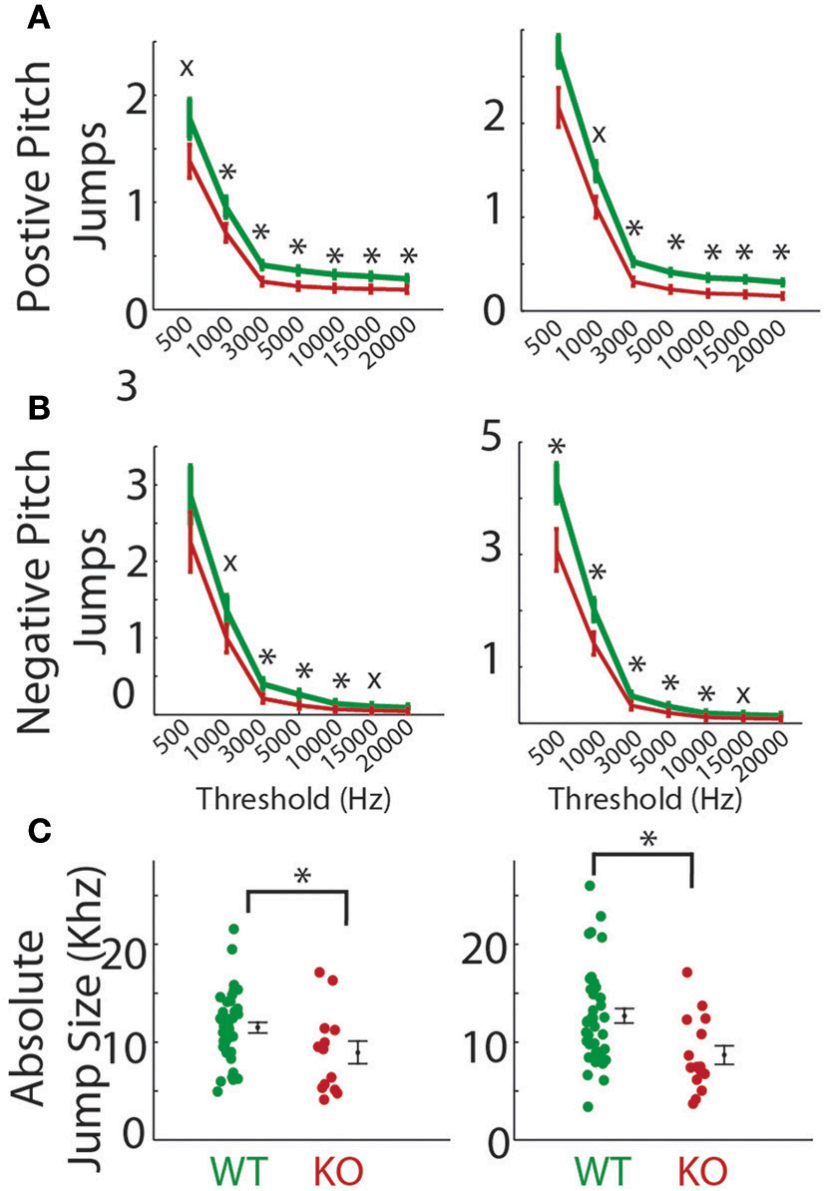
**Pitch jumps of calls in the ***Gnptab***^**−/−**^ pups compared to the ***Gnptab***^**+/−**^ and ***Gnptab***^**+/+**^ pups. (A)** Mean number of jumps per call given a criterion for the minimum size of jump (in Hertz, x-axis). Data from pups on P5 (left) and P8 (right) for jumps going up in pitch (positive), and **(B)** jumps going down in pitch. **(C)** Size of maximum pitch jump per call on P5 (left) and P8 (right) (*Gnptab*^+/+^ pups (green), and *Gnptab*^−/−^ pups (red). Each dot represents the mean of one animal. There was a significant difference between *Gnptab*^+/+^ calls and *Gnptab*^−/−^ calls on P5 (*t*-test; *p* = 0.0373) and P8 (*t*-test; *p* = 0.0056). Asterisks denote *p* < 0.05, x denotes a *p* < 0.1.

Abrupt pitch jumps are a naturally occurring feature of mouse vocalizations as can be seen in Figure 1B (Liu et al., 2003; Holy and Guo, 2005). The incidence of pitch jumps were significantly reduced in the *Gnptab*^−/−^ pup calls compared to the *Gnptab*^+/+^ pup calls on both P5 and P8 over a wide range of criteria used to define the minimum size pitch jumps for both positive and negative jumps (Figures 6A,B; Supplemental Table 2). Likewise the magnitude of the largest pitch jump in each call was also decreased in the *Gnptab*^−/−^ pup calls compared to *Gnptab*^+/+^ pups on P5 (WT = 11485 ± 550 Hz, KO = 8947 ± 1141 Hz; *t*-test; *t* = 2.139, *p* = 0.0373), and P8 (WT = 12689 ± 746 Hz, KO = 8687 ± 953 Hz, *t*-test; *t* = 2.886, *p* = 0.0056; Figure 6C).

### Temporal difference and entropy

**Figure 7:**
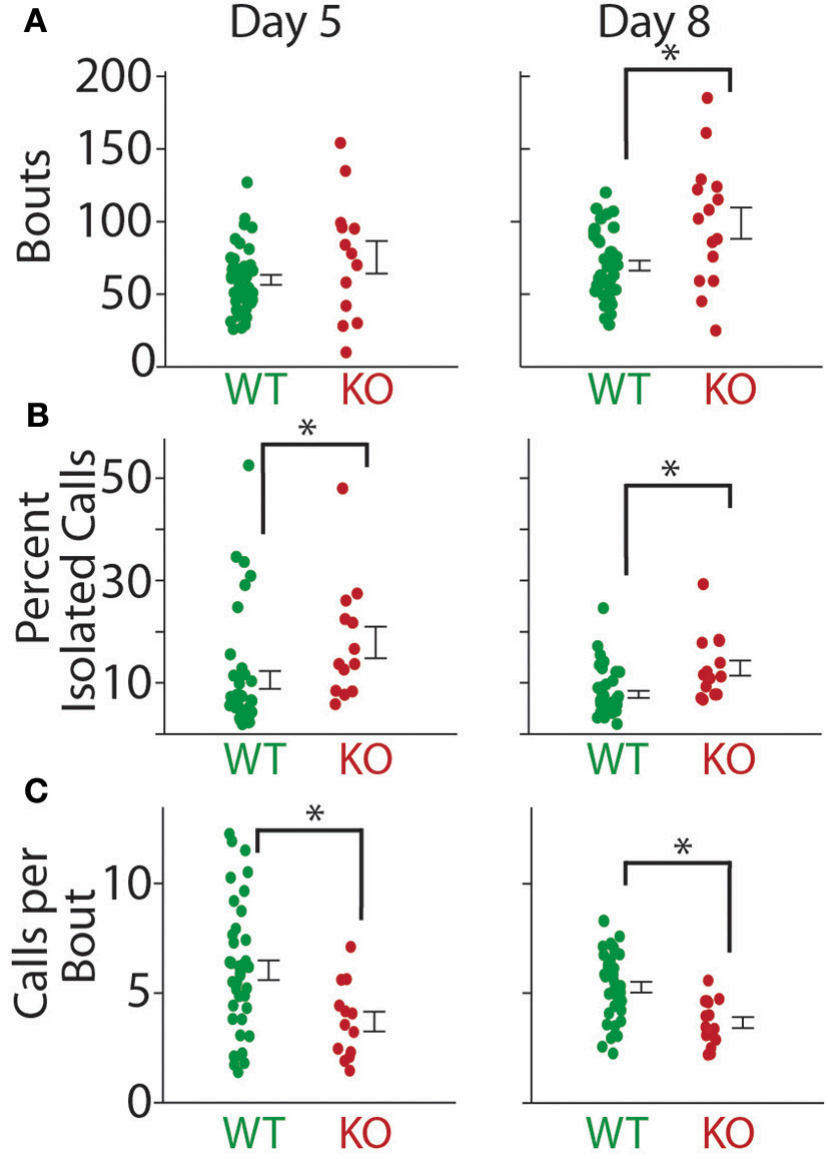
**Bout structure of calls in ***Gnptab***^**+/+**^, and ***Gnptab***^**−/−**^ animals differed. (A)** Mean number of bouts emitted on P5 (left) and P8 (right). There was a significant increase in the number of bouts in the *Gnptab*^−/−^ (red) compared to the *Gnptab*^+/+^ (green) by P8 (*t*-test; *p* = 0.0016). Each dot represents the mean of one animal. **(B)** Same as in A but for percent of isolated calls. There was a significant increase in the *Gnptab*^−/−^ compared to the *Gnptab*^+/+^ for P5 (*t*-test; *p* = 0.045) and P8 (*t*-test; *p* = 0.001). **(C)** Same as in A but for calls per bout. There was a significant decrease in the *Gnptab*^−/−^ mice compared to the *Gnptab*^+/+^ mice for P5 (*t*-test; *p* = 0.007) and P8 (*t*-test; *p* = 0.0005). Bars indicate the standard error of the mean. Asterisks denote *p* < 0.05.

The mean number of bouts per recording (P8; WT = 69.63 ± 3.45, KO = 98.93 ± 10.76, *t*-test; *t* = 3.326, *p* = 0.0016) increased significantly in the *Gnptab*^−/−^ pups compared to the *Gnptab*^+/+^ pups by P8 (Figure 7A). This was at least in part due to a significant increase in the percent of isolated calls (i.e., calls surrounded by inter-bout pauses) per recording (P8; WT = 7.8% ± 0.7%, KO = 12.9% ± 1.5%, *t*-test *t* = 3.475, *p* = 0.001; Figure 7B). The tendency of the vocalization data recorded on P5 was similar to that of P8 for the mean number of bouts per recording (P5; WT = 59.83 ± 3.50, KO = 75.31 ± 11.20, *t*-test; *t* = 1.714, *p* = 0.092), though not significant. On P5, the percentage of isolated calls differed (P5; WT = 10.6% ± 1.7%, KO = 17.9% ± 3.1%, *t*-test; *t* = 2.051, *p* 0.045). Relatedly, on both P5 and P8 of recording, the *Gnptab*^−/−^ pups had significantly fewer calls per bout when compared to the *Gnptab*^+/+^ pups (P5, WT = 6.04 ± 0.449, KO 3.69 ± 0.45; *t*-test; *t* = 2.776 *p* = 0.007; P8, WT = 5.28 ± 0.243, KO = 3.66 ± 0.26; *t*-test; *t* = 3.678, *p* = 0.0005); Figure 7C).

**Figure 8:**
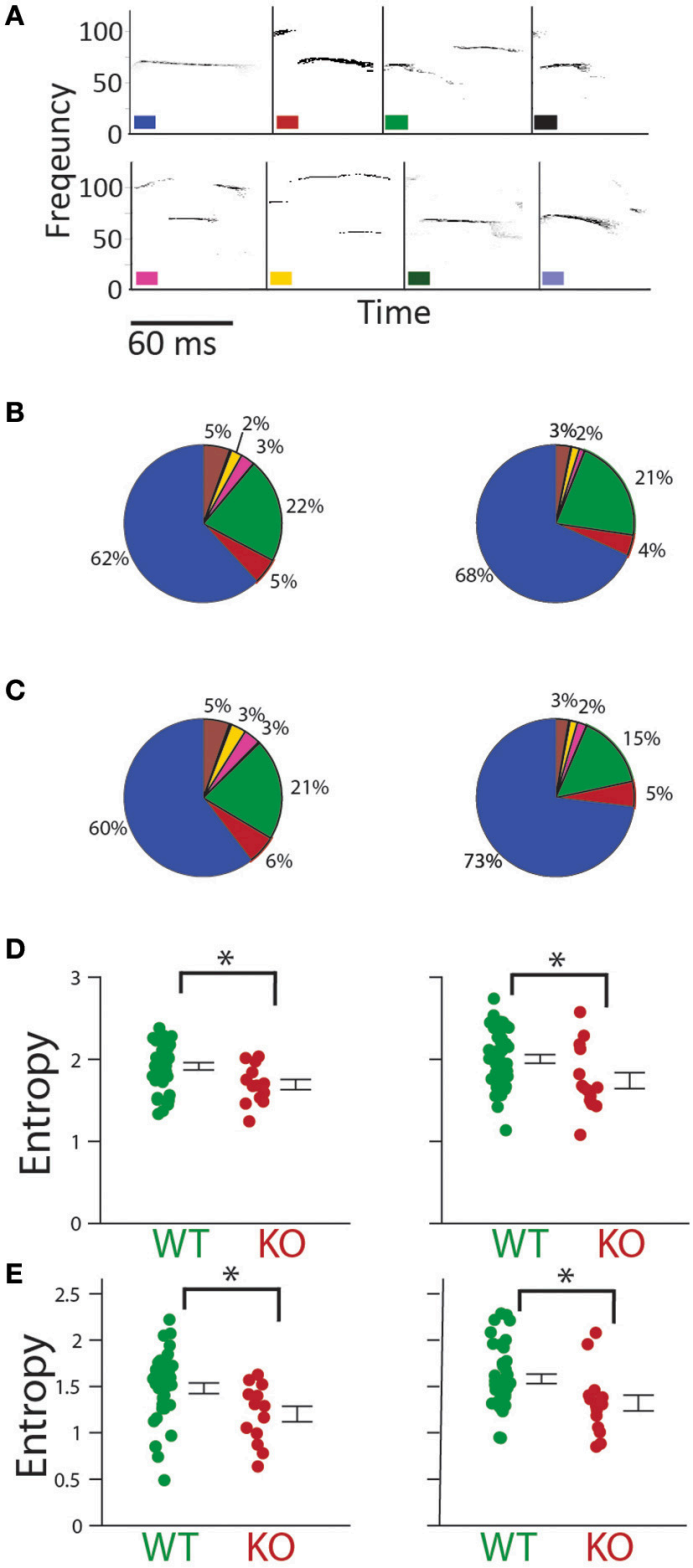
**Type and temporal sequencing of calls. (A)** Syllable identification scheme showing examples of each type of syllable. **(B)** Percentage of each syllable type in *Gnptab*^+/+^ (left), and *Gnptab*^−/−^ mice (right) in P5 and **(C)** P8. Each color represents one syllable type. **(D)** Entropy of syllable type usage was significantly decreased in the *Gnptab*^−/−^ mice (red) compared to the *Gnptab*^+/+^ (green) on P5 (*t*-test; *p* = 0.012) and P8 (*t*-test; *p* = 0.018). **(E)** *Gnptab*^−/−^ mice also showed a decrease in the temporal sequence entropy, using a first order Markov process, on P5 (*t*-test; *p* = 0.017) and P8 (*t*-test; *p* = 0.013). Bars indicate the standard error of the mean. Asterisks denote *p* < 0.05.

Next, calls were classified based on a previously published classification (Liu et al., 2003; Barnes et al., 2016) (Figure 8A). The breakdown of call type is shown in Figures 8B,C. Entropy of call type usage was calculated from the proportion of different call types (see Methods). *Gnptab*^−/−^ call type entropy was significantly decreased in both P5 (P5; WT = 1.915 ± 0.044, KO = 1.692 ± 0.062 *t*-test; *t* = 2.593, *p* = 0.012); and P8 (P8; WT = 2.004 ± 0.053, KO = 1.742 ± 0.098 *t*-test; *t* = 2.443 *p* = 0.018 Figure 8D). We also looked at the diversity of call type sequences by analyzing the entropy in the first-order Markov process. We found this too to be significantly decreased in the *Gnptab*^−/−^ mice on both days (P5; WT = 1.480 ± 0.057, KO = 1.202 ± 0.084 *t*-test; *t* = 2.459, *p* = 0.017; P8, WT = 1.582 ± 0.052, KO = 1.321 ± 0.085; *t*-test; *t* = 2.573, *p* = 0.013); Figure 8E).

### Inferring genotype from vocal abnormalities

So far, group differences have been identified in the different genotypes. Next we asked whether using these features we can identify a particular recording as coming from either a *Gnptab*^+/+^ or *Gnptab*^−/−^ mouse. We developed an automated classification scheme using the following variables: normalized intra-bout pause duration, largest pitch jump per call, bouts per recording, calls per bout, and percent isolated calls. The number of animals in each data set was first matched. Next, the data was projected into 5 dimensional space. Individual recordings were then classified as *Gnptab*^−/−^ or *Gnptab*^+/+^ based on the majority identity of its 3 nearest neighbors, leaving the recording itself out of the comparison set. We found that a larger number of recordings could be correctly classified compared to chance on P8 (80%; c.i., 67–90%**)**. Results were similar for classifications based on 1, 3, and 5 nearest neighbors.

### Heterozygous *Gnptab*^+/−^ pups

**Figure 9:**
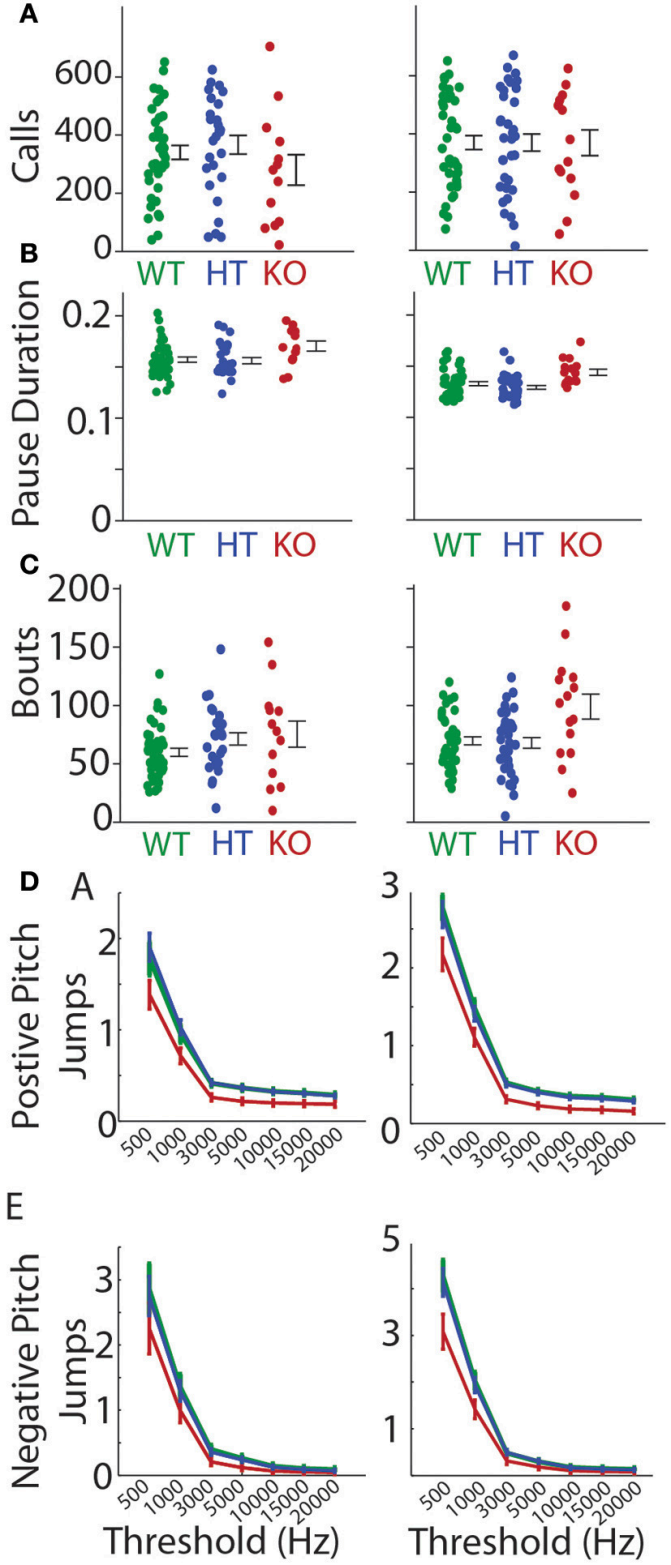
**Heterozygous ***Gnptab***^**+/−**^ mice were similar to wildtype mice**. Heterozygous *Gnptab*^+/−^ (HT, blue) mice did not differ from *Gnptab*^+/+^ mice on P5 (left) or P8 (right) for **(A)** Call duration **(B)** Intra- bout pause durations, **(C)** Bouts per recording, **(D)** Upward (positive) jumps or **(E)** Downward (negative) jumps.

We examined the features of the heterozygous pup calls (*Gnptab*^+/−^). In all of the features we analyzed *Gnptab*^+/−^ were not significantly different from that of wild type *Gnptab*^+/+^ mice. Likewise, features that were significantly different between *Gnptab*^+/+^ mice and *Gnptab*^−/−^ homozygous knockout mice, were also significantly different between *Gnptab*^+/−^ heterozygous mice and *Gnptab*^−/−^ homozygous mice in all but two cases (call duration on P8 and percent isolated syllables on P5, Supplemental Table 1, Figure 9).

## Discussion

### Summary and interpretation of findings

We examined the isolation calls of *Gnptab*^−/−^ pups and compared them with the isolation calls of their wild type littermates, *Gnptab*^+/+^. *Gnptab*^−/−^ mice cannot make the first enzyme in the pathway that targets lysosomal enzymes to their lysosomes. Instead, in these mice, lysosomal enzymes end up in the extracellular space (Gelfman et al., 2007; Idol et al., 2014; Paton et al., 2014).

In adult *Gnptab*^−/−^ mice, many deficits— physical, histological, and behavioral—have been documented (Gelfman et al., 2007; Idol et al., 2014; Paton et al., 2014). Fewer abnormalities have been identified for younger mice. Physically, studies have found *Gnptab*^−/−^ mice to have a normal weight at 2 weeks and 4 weeks. By 6 weeks (Gelfman et al., 2007)—8 weeks (Paton et al., 2014) *Gnptab*^−/−^ showed a significant decrease in their weight compared to *Gnptab*^+/+^ mice. Histological abnormalities have been found at 1 month of age including *Gnptab*^−/−^ mice having an increase in the number of cells undergoing apoptosis (Gelfman et al., 2007). Behaviorally, 1 month *Gnptab*^−/−^ mice preform normally on a 1 h locomotor test but show deficiencies on 3/7 locomotor assays (Idol et al., 2014). Here we find abnormalities both in weight and vocalizations as early as P5. As early as P5, we found a trend (*p* = 0.09) for *Gnptab*^−/−^ pups to weigh less. By P8 this difference was significant. The difference between the weights found in previous studies and our study, could be due to variety of factors including statistical power (Gelfman et al., 2007) or background strain differences (Paton et al., 2014).

Behaviorally, we found that the vocalizations of *Gnptab*^−/−^ mouse pups were grossly normal. *Gnptab*^−/−^ pups had similar call rates (both in the number of calls and number of clicks per recording) and call volumes. *Gnptab*^−/−^ pups were able to make both high and low frequency calls. It appears that the vocal tract is intact capable of making the same repertoire of vocalizations.

Many features of the *Gnptab*^−/−^ isolation calls were distinct from *Gnptab*^+/+^. Duration of the mouse pup isolation calls in the *Gnptab*^+/+^, and *Gnptab*^−/−^ animals differed significantly. The intra-bout pause duration was increased in the *Gnptab*^−/−^ animals though this could be due to the weight differences between the two groups as this measure was always significantly correlated with the weight of the pup. The difference in weight of *Gnptab*^−/−^ may be causing the difference in USVs, or the mutation may independently cause the difference in weight and difference in USVs.

Pitch jumps sizes were markedly different between genotypes, with *Gnptab*^−/−^ pups showing significantly smaller and fewer abrupt pitch jumps. Overall, the *Gnptab*^−/−^ pups were not quieter than their wild-type litter mates, but they did have less power in the higher frequencies. This fits well with having smaller and fewer pitch jumps. Though there we no difference in the number of calls emitted during the recordings, the number of bouts were greater in the *Gnptab*^−/−^ pups compared to *Gnptab*^+/+^ pups. This was in part due to the increase in isolated calls observed in the *Gnptab*^−/−^ recordings. *Gnptab*^−/−^ pups were physically able and often did string many calls together, but their vocalizations more often included single calls. Entropy both for the proportion of different call types and the entropy of the temporal sequencing were decreased in the *Gnptab*^−/−^ mouse pup calls. This may be in part due to the decrease in pitch jumps, a defining feature of many call types.

Heterozygous pups *Gnptab*^+/−^ were not significantly different, in any of the features examined, from wild-type littermates *Gnptab*^+/+^. This fits well with human findings, as humans with one copy of the *GNPTAB* gene missing are generally phenotypically normal.

### Abnormalities in ultrasonic vocalizations over time

By P8, the *Gnptab*^−/−^ pups showed many abnormalities compared to the *Gnptab*^+/+^ pups. In the cases where recording from P5 pups did not show a statistically significant difference between *Gnptab*^+/+^ and *Gnptab*^−/−^ animals, the recordings often exhibited the same general trend, suggesting the differences were growing as time passed. It is possible that some of the difference found in the *Gnptab*^−/−^ pups was due to a maturation delay, rather than a long term deficiency. Indeed the mean call length on the *Gnptab*^−/−^ pups calls on P8 (0.046 s) was similar to that of the *Gnptab*^+/+^ pup calls on P5 (0.044 s). A more longitudinal study might be able to determine if juvenile and adult vocalizations are similarly affected.

### Comparison to literature

The speech abnormalities in patients with Mucolipidosis II/III have not been well-quantified. Otomo et al found that, out of 13 patients diagnosed with Mucolipidosis II, 12 were unable to utter single words (Otomo et al., 2009).

These data can be compared to data recently published on pup isolation calls in the *Gnptab*^mut/mut^ mice (Barnes et al., 2016). These mice were engineered to carry a homozygous Glu1179Lys mutation in *Gnptab* homologous to the Glu1200Lys mutation in human *GNPTAB* well characterized for its role in stuttering (Kang et al., 2010; Fedyna et al., 2011). *Gnptab*^−/−^ pups showed more varied abnormalities and in features of their calls than the *Gnptab*^mut/mut^ mice. *Gnptab*^mut/mut^ showed a difference in the number of vocalizations per unit time, while the *Gnptab*^−/−^ were normal for this measure.

Both modifications had effects on the temporal structure of the calls as well. They caused a decrease in first order entropy of the call sequences. Pauses between calls were also affected in both cases. In the knockout, the intra-bout pause duration was affected while the missense mutation increased the occurrences of longer pauses. Both modifications resulted in an increase in the number of isolated syllables. The pathways by which these modifications can affect such specific features of mouse vocalizations are unknown, as is the reason why the knockout fails to affect the number of calls and other features perturbed by the missense mutation.

These data show that mutations in the lysosomal enzyme targeting pathway affect mouse pup isolation calls. Persons who suffer from Mucolipidosis II also have speech abnormalities, suggesting that in regards to vocalizations, this mutation affects the vocalizations of both species from a very young age.

## Methods

Mice were donated by the Stuart Kornfeld laboratory. Mice were generated by the OmniBank gene trap library (Zambrowicz et al., 1998; Zambrowicz and Sands, 2003). Mice used in this study were of mixed genetic background (129/SvEvBrd and C57BL/6J) and hence all comparisons employed littermate controls. Oligonucleotide wild-type primers (WT 3′: GAGAATGCACAC GCTGATGGGGCCCATTCA WT 5′: GCCCATTCA TTTCTGACCTGCTCATACCCC) and NEO primers (Neo 3′; CGCCAAGCTCTTCAG CAATATCACGGG TAG, Neo 5′ TGCTC CTGCCGAGAAAGTATCCATCATGGC) were used in two separate reactions to amplify corresponding wild-type and mutant *GNPTAB* alleles.

Mice were kept on a 12 h light dark cycle, tested during the light part of the cycle. Mice received standard chow and water *ad libitum*. To generate test subjects, heterozygous mice were crossed. Breeding cages were set up with one male and two females. Females were separated from all other mice before they gave birth. Mice came from 18 liters. The first day that a litter was discovered (dams were checked for pups daily) was considered as postnatal day zero. We recorded from mice on postnatal P5 (P5) and 8 (P8), days that are in the peak pup vocalization. Altogether there were 101 pups. (The average size of litters was 5.6 pups.) Of these, weight measurements were taken for 83 of the mice, and correlations were performed to vocalization parameters (see weights in Figure 5). For recordings, the dam was removed from the cage and placed in a new clean cage away from the home cage. The home cage with pups was placed in an incubator set at 34 degrees. Before testing began, the auxiliary temperature of the pups was taken with a flexible thermistor on the back of the animal (temperature probe from Omega Engineering Inc.) Approximately 10 min after the pups were isolated from the dam, each pup was separately placed carefully into the test chamber. Recordings lasted 3.5 min. Afterwards, the pup was weighed, tattooed and a small piece of tail was taken for genotyping. The pup was then returned to the dam (Hofer et al., 2001). Mice that did not have at least 10 calls were not included in the analysis. One heterozygous and one wild type were dropped because of this criterion.

The testing equipment was described previously (Barnes et al., 2016). Recording occurred in a wooden enclosure (to attenuate external sounds) with a transparent Plexiglas front measuring 33 l × 20.3 w × 16.8 h centimeters. Sounds were digitized at 250 KHz with 16 bit resolution (National Instruments, Austin, Texas, United States). The microphone (1/4” microphone, model 4939, Brüel and Kjær, Nærum, Denmark) was suspended from the top of the cage approximately 5 centimeters from the bottom of the recording box.

### Statistics and analysis

All analyses were done using in-house MATLAB programs, some of which are available online at http://holylab.wustl.edu/. Waveforms were pre-processed, band-pass filtered (25–110 kHz), and calls identified using mean frequency, “spectral purity” (fraction of total power concentrated into a single frequency bin), and the “spectral discontinuity” (the change in the allocation of power across frequencies between two adjacent time bins) (Barnes et al., 2016). Stored acoustical waveforms were processed using MATLAB to compute the sonogram (512 samples/block, half-overlap, resulting in a time resolution of 1.02 ms and a frequency resolution of 0.98 kHz). Clicks were defined as milliseconds where fewer than 200 of the 512 samples were empty.

Analysis code implemented a fully-automated algorithm and was therefore blind to genotype. To calculate the number of calls and the duration of calls and pauses, each vocalization or pause contributed to the mean for each animal or subject; each individual's mean was then averaged to obtain the group average. A *t*-test was then performed to compare groups with each individual's mean. All *t*-tests were two-tailed. An alternative analysis, where each call contributes to the overall mean was also performed and produced comparable results, as shown.

For the call analysis, the definitions set forth in Arriaga et al. were used, with the exception of classes “i, j, k” these call types were grouped into the category “other” (Liu et al., 2003; Barnes et al., 2016). This classification was done using a fully-automated algorithm.

Bout-level analyses defined bouts based on histograms of pause lengths for all groups of mice in each day we recorded. Histograms were constructed with a range of bin sizes (50 to 300 ms). The middle of minimum bin in the range of 0.15–0.32 s was averaged across all bin sizes to determine the criteria for an inter-bout pause. The resulting minimum intra-bout/inter-bout cutoff was determined to be 0.273 s in P5 and 0.239 s in P8 recordings.

Entropy of call usage was calculated from the proportion of different call types; entropy for the temporal sequence (modeled as a first-order Markov process) was given by *H*2 = −Σ*p*(X) Σ*p* (X|Y) log2*p* (X|Y) with X and Y being each call type (Ey et al., 2013).

For the classification of genotype analysis, data were first normalized. A random sample of *Gnptab*^+/+^ recordings were chosen to match the n of the *Gnptab*^−/−^ recordings. We then categorized each recording as being either from either a *Gnptab*^+/+^, mouse or Gnptab^−/−^ mouse based on nearest neighbors. Choices of 1, 3, and 5 closest neighbors all yielded very similar results. This analysis was performed 10,000 times each time selecting a random sample of *Gnptab*^+/+^ recordings.

To calculate the power spectrum, all time periods during a call were grouped and then the power spectrum was calculated per subject. The subjects' averages were then bootstrapped 1000 times to determine the 95% confidence intervals. We reported as significant, significant bins adjacent to at least one other significant bin.

For each call, the number of pitch jumps was determined. Jumps were defined as abrupt changes in pitch exceeding a minimum threshold. We explored thresholds of 500, 1000, 3000, 5000, 10,000, 15,000, and 20,000 Hz, and found that the significance of the results was not dependent upon the particular choice of threshold. Pitch was defined as the dominant frequency as a function of time, discarding periods of dropouts (Barnes et al., 2016). P values for Figure 6 can be found in Supplemental Table 1. In a separate measurement, the size of the largest pitch jump in each call was determined and then averaged across calls for each animal. We also tested for effects of gender and found no significant effect of gender on phenotype for any of the features studied.

A regression analysis was done of wild-type pups for each of our parameters to determine if the parameters directly correlated with weight. The *Gnptab*^−/−^ and *Gnptab*^+/−^ pups were not included because the degree that each pup was affected could be correlated with both the weight of the animal and the severity of the deficiency. In fact several of the parameters for the knockout did correlate with body weight (Supplemental Figure 2).

## Ethics statement

This study was approved by the Animal Studies Committee at Washington University in St. Louis (IACUC equivalent). Washington University protocol 20130179.

## Author contributions

TB and TH: Designed the experiments; TB: Performed the experiments; TB and TH: analyzed the data and wrote the paper.

## Funding

This work was supported by the following grant: National Institutes of Health Pioneer's Grand DP1 0D006437.

### Conflict of interest statement

The authors declare that the research was conducted in the absence of any commercial or financial relationships that could be construed as a potential conflict of interest.
