# Supplementary material for: Knockout of Lysosomal Enzyme-Targeting Gene Causes Abnormalities in Mouse Pup Isolation Calls
Source: Front Behav Neurosci. 2017 Jan 4;10:237. doi: 10.3389/fnbeh.2016.00237 (PMC5209381; doi:10.3389/fnbeh.2016.00237)
Supplement: Supplementary file 1 [file DataSheet1.docx]

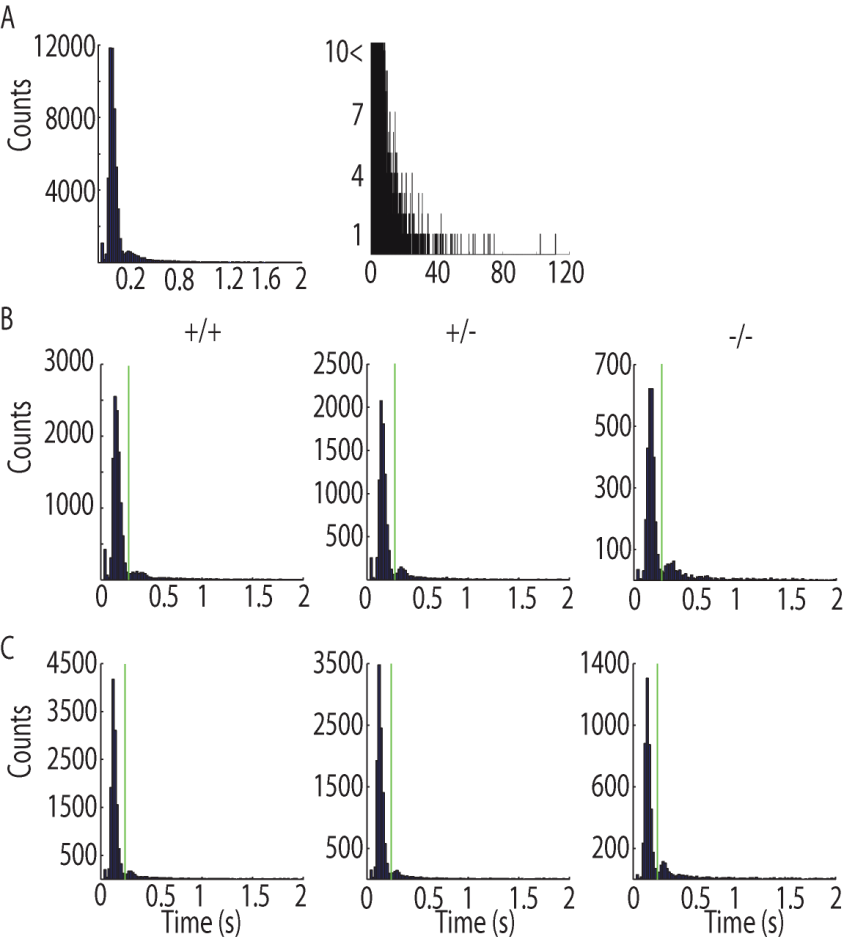


**Supplemental Figure 1**. Distribution of pause durations. (A) Histogram of all pauses for all genotypes on both P5 and P8. Right) a zoom in on the y-axis in conjunction with a zoom in on the y-axis showing the long tail of the distribution. (B) Histograms for *Gnptab*^+/+^ (left), *Gnptab* ^+/-^ (middle), and *Gnptab* ^-/-^ (right) on P5 and (C) P8. Green bars indicate intra-bout/inter-bout calculated cutoff.

**Supplemental Table 1**

Comparison of *Gnptab* ^+/-^ with *Gnptab* ^+/+^ and *Gnptab* ^-/-^ mice on P5 and P8. Note that due to multiple comparisons, the Bonferroni .05 equivalent is .017 (three groups).

|  | Day 5 | | | Day 8 | | |
| --- | --- | --- | --- | --- | --- | --- |
|  | WT/HT | HT/KO | WT/KO | WT/HT | HT/KO | WT/KO |
| Call Number | 0.511 | 0.159 | 0.254 | 0.993 | 0.993 | 0.997 |
| Click | 0.978 | 0.540 | 0.416 | 0.271 | 0.529 | 0.291 |
| Duration of Call | 0.672 | 0.224 | 0.126 | 0.188 | 0.257 | 0.024 |
| Duration of Pause | 0.822 | 0.097 | 0.084 | 0.315 | 0.709 | 0.397 |
| Inter-Bout Pause Duration | 0.417 | 0.193 | 0.197 | 0.270 | 0.383 | 0.334 |
| Intra-Bout Pause Duration | 0.827 | 0.016 | 0.018 | 0.202 | 0.0001 | 0.006 |
| Power | 0.338 | 0.345 | 0.076 | 0.240 | 0.963 | 0.292 |
| Number of Bouts | 0.070 | 0.714 | 0.092 | 0.752 | 0.003 | 0.0016 |
| Percent Isolated Calls | 0.638 | 0.114 | 0.045 | 0.452 | 0.00008 | 0.001 |
| Mean Calls per Bout | 0.154 | 0.036 | 0.007 | 0.857 | 0.0002 | 0.0005 |
| Maximum Mean Pitch Jump | 0.480 | 0.048 | 0.037 | 0.557 | 0.001 | 0.006 |
| Entropy of Call Type | 0.330 | 0.003 | 0.012 | 0.447 | 0.004 | .0178 |
| Entropy of Sequence of Call Type (H2) | 0.495 | 0.009 | 0.017 | 0.431 | 0.005 | 0.013 |

Supplemental Table 2

T-test P value of the mean number of pitch jumps for *Gnptab*^+/+^ and *Gnptab* ^-/-^.

|  | Positive Jumps | | Negative Jumps | | Absolute Jumps | |
| --- | --- | --- | --- | --- | --- | --- |
| Minimum Size of Pitch Jump (Hertz) | P5 | P8 | P5 | P8 | P5 | P8 |
| 500 | 0.0771 | 0.1 | 0.1494 | 0.0012 | 0.0897 | 0.0064 |
| 1000 | 0.049 | 0.0883 | 0.0771 | 0.0013 | 0.0499 | 0.0085 |
| 3000 | 0.0064 | 0.0029 | 0.0054 | 0.0015 | 0.0033 | 0.0011 |
| 5000 | 0.0053 | 0.0016 | 0.0011 | 0.004 | 0.0012 | 0.001 |
| 10000 | 0.0096 | 0.0046 | 0.0105 | 0.044 | 0.0038 | 0.0062 |
| 15000 | 0.0147 | 0.0051 | 0.0558 | 0.0927 | 0.0076 | 0.0099 |
| 20000 | 0.0352 | 0.0049 | 0.1237 | 0.1052 | 0.0195 | 0.0102 |


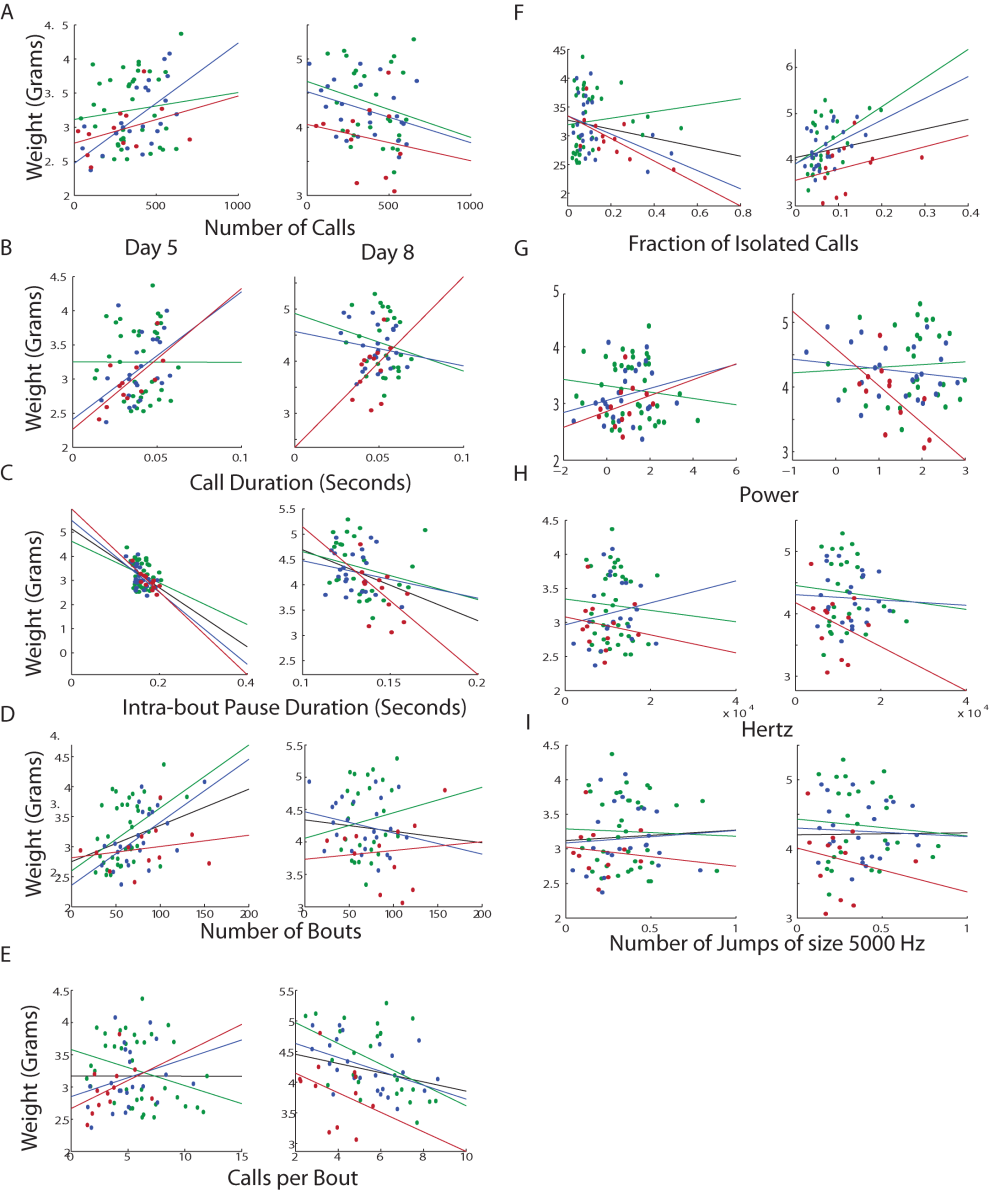


**Supplemental Figure 2.** Correlation between the weight of the animal and parameters studied on P5 (left) and (right) of *gnptab* +/+ (WT; green, P5 n=37, P8 n=27) *gnptab* +/- (HT; blue P5 n=23, P8 n=24) and *gnptab* -/- (KO; red, P5 n=12, P8 n=12). Black lines indicate the correlation of all animals regardless of genotype. A) Number of Calls B) Call duration. C) Length of intra-bout pauses. D) Number of Bouts. E) Mean number of calls per bout. F) Percentage of isolated calls G) Power. H) Mean Maximum frequency jump in Hertz. I) Mean number of pitch jumps that were at least 5000 Hertz in magnitude.
